# Two Birds, One Stone: Simultaneous LAA Occlusion and Lateral Mitral PVL Closure

**DOI:** 10.1016/j.jaccas.2026.108588

**Published:** 2026-06-03

**Authors:** Yu-Chuan Chuang, Ming-Ju Chuang, Tsun-Jui Liu

**Affiliations:** a Department of Cardiovascular Center, Taichung Veterans General Hospital, Taichung, Taiwan; b National Chung-Hsing University School of Medicine, Taichung, Taiwan; c National Yang-Ming-Chiao-Tung University School of Medicine, Taipei, Taiwan; d Pingtung Veterans General Hospital, Pingtung, Taiwan

**Keywords:** chronic heart failure, imaging, mitral valve, valve replacement

**Figure:**
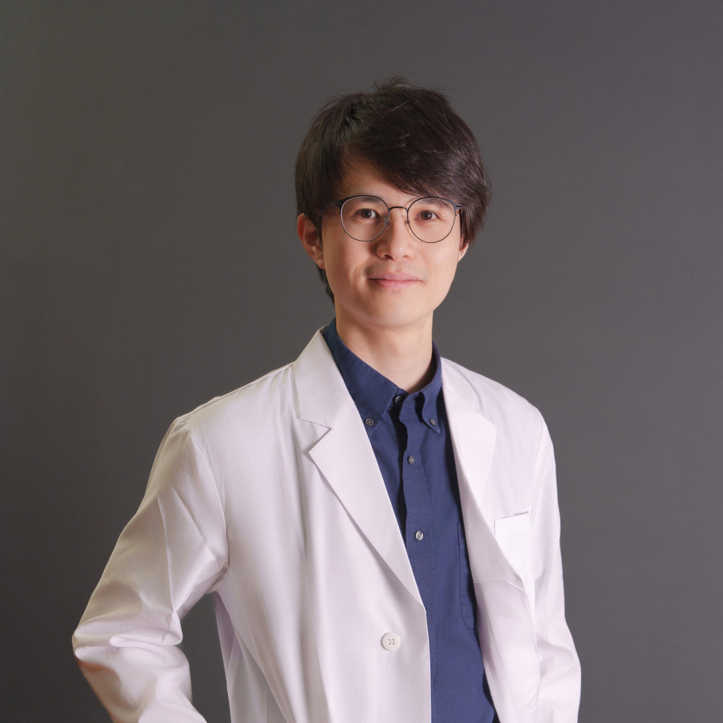

Structural heart disease interventions are undergoing a paradigm shift from isolated, single-lesion treatments to comprehensive, patient-tailored anatomical strategies that address the full spectrum of cardiac pathology.^1^ Unlike surgery, which typically addresses all lesions simultaneously, transcatheter interventions allow staged approaches that allow clinicians to assess the effect of correcting the principal valve lesion on the remaining pathology, optimizing the timing of subsequent interventions.^2^ Advanced imaging modalities, including cardiac computed tomography, 3-dimensional echocardiography, and computational modeling, enable precise anatomical assessment and procedural planning tailored to individual patient anatomy.^3^^,^^4^ The feasibility of comprehensive percutaneous approaches has been demonstrated in complex cases that combine coronary intervention, transcatheter aortic valve replacement, mitral repair, and closure of the left atrial appendage (LAA) in staged fashion.

In this issue of *JACC: Case Reports*, Kay et al^5^ elegantly illustrate this evolving paradigm by presenting a novel “2-for-1” transcatheter approach.^5^ The authors describe the case of an 81-year-old man with 2 prior mitral valve replacements who presented with recurrent, symptomatic paravalvular leak (PVL). The patient's clinical path was severely complicated by recurrent decompensated heart failure and hemolytic anemia that required transfusions. Furthermore, he had a high thromboembolic risk driven by paroxysmal atrial fibrillation (CHA_2_DS_2_-VASc score of 7), but his ability to tolerate uninterrupted oral anticoagulation was severely limited by gastrointestinal arteriovenous malformations. Faced with prohibitive surgical risk and complex competing comorbidities, the operators used a single Watchman FLX Pro device to simultaneously occlude the LAA and seal an adjacent lateral mitral PVL.

## The Anatomical Rationale: Proximity Is Key

The standard percutaneous management of multiple PVLs often requires complex, tortuous trajectories to deploy multiple vascular plugs.^6^ The successful use of 12-mm Amplatzer Vascular Plug II devices for medial and posterior defects demonstrates the standard percutaneous technique, while the lateral PVL's “unique anatomical opportunity” suggests a more favorable access route or defect morphology that may have allowed alternative approaches.^7^

Because the lateral mitral annulus is located in close spatial proximity to the LAA ostium, the operators hypothesized that the structural footprint of an LAA occlusion (LAAO) device could be used to provide a dual seal.^8^^,^^9^ The strategic deployment of a 31-mm Watchman FLX Pro device within the appendage allowed the proximal face of the occluder to extended sufficiently to cover the lateral PVL. This single-device strategy effectively reduced the regurgitant jet to trace, abolished retrograde pulmonary vein systolic flow, and mitigated the need for additional occluders in the lateral space.^5^

## The Crucial Role of Preprocedural Imaging

The success of this comprehensive, single-device approach is inextricably linked to meticulous preprocedural planning. The authors rightly highlight the integration of preprocedural cardiac computed tomography and intraprocedural 2-dimensional/3-dimensional transesophageal echocardiography.^5^ This multimodal imaging analysis was the cornerstone that allowed the structural heart team to identify the exact spatial relationship between the LAA ostium and the persistent lateral PVL.^10^

When attempting a simultaneous closure, precise spatial measurements are vital.^11^ The distance between the ostium and the paravalvular defect must be carefully assessed to ensure that the proximal disc or fabric footprint possesses a sufficient radius to cover the leak without impinging on the mechanical or bioprosthetic valve components.^9^^,^^12^ These critical requirements are summarized in Table 1.

**Table 1:** Anatomical Considerations and Imaging Prerequisites for Simultaneous LAAO and Lateral Mitral PVL Closure

Anatomical Parameter	Ideal Prerequisite for Simultaneous Closure	Primary Imaging Modality	Clinical Consequence If Unmet
PVL location	Strictly lateral or anterolateral (eg, 9-11 o'clock position on the mitral surgical annulus).	3D TEE/cardiac CT	Device disc will not cover the defect; alternative approaches (eg, transseptal/apical specific PVL plugs) required.
LAA ostium to PVL distance	Very close proximity. The distance must be less than the radius of the LAAO device's proximal disc/fabric footprint.	Cardiac CT/3D TEE	Residual significant paravalvular regurgitation due to incomplete coverage by the device disc.
LAA landing zone depth and morphology	Adequate depth and favorable lobe morphology to ensure secure anchoring of the distal device component.	Cardiac CT/TEE/fluoroscopy	Device embolization or micro-dislodgement caused by the mechanical stress of sealing the adjacent annulus.
Prosthetic valve clearance	Sufficient distance between the LAA ostium/annulus and the prosthetic valve mechanism (especially mechanical hinges).	Cardiac CT/3D TEE	Mechanical leaflet impingement or bioprosthetic leaflet restriction by the protruding LAAO disc.
LCx trajectory	LCx course in the AV groove must be safely distanced or protected from the expansive force of the LAAO device.	Cardiac CT/coronary angiography	LCx compression leading to acute myocardial ischemia or infarction.

3D = 3-dimensional; AV = atrioventricular; CT = computed tomography; LAA = left atrial appendage; LAAO = left atrial appendage occlusion; LCx = left circumflex artery; PVL = paravalvular leak; TEE = transesophageal echocardiography.

## Procedural Caveats and Future Perspectives

Although intellectually and procedurally elegant, this technique requires highly specific “Goldilocks” anatomy and is not universally applicable to all mitral PVLs. For covering the lateral PVL, the oversizing percentage should be carefully decided according to pre-LAAO image evaluation. The interaction between the LAAO and circumflex artery should be taken into account, as this has happened in previous studies.^13^^,^^14^

Furthermore, operators must be prepared for the possibility of an incomplete “dual seal.” As Kay et al^5^ outline in their procedural pitfalls, if significant residual lateral PVL persists after the initial deployment of the Watchman device, operators must be prepared to recapture and reposition the device to optimize ostial coverage while simultaneously maintaining adequate LAA compression and seal.^5^ If the single-device strategy ultimately fails to seal the PVL, the operator should pre-cross the lateral PVL with an angled Glidewire before final Watchman release, maintaining a stable rail to deploy a dedicated vascular plug.

## Conclusions

To our knowledge, this report by Kay et al^5^ represents the first documented application of a Watchman device for the closure of a mitral PVL. It exemplifies how a profound understanding of patient-specific structural anatomy, guided by advanced multimodality imaging, can foster innovative, off-label device applications. As the field of structural heart disease continues to mature, finding synergistic solutions to concomitant pathologies will become increasingly important. For high-risk patients battling both atrial fibrillation and complex valvular disease in the setting of severe bleeding limitations, this “2-for-1” strategy offers a highly effective treatment paradigm that simplifies the procedural approach and minimizes the need for multiple high-risk interventions.

## Funding Support and Author Disclosures

The authors have reported that they have no relationships relevant to the contents of this paper to disclose.
